# Personalized GenAI coaching and self-regulation in L2 writing across a nine-week active-control study

**DOI:** 10.3389/fpsyg.2026.1904842

**Published:** 2026-10-14

**Authors:** Bo Yuan

**Affiliations:** School of Educational Studies, Universiti Sains Malaysia, Penang, Malaysia

**Keywords:** adaptive learning, learning analytics, metacognitive prompting, personalized GenAI coaching, self-regulated learning

## Abstract

**Introduction:**

Personalized rationale is an explanation of why a metacognitive strategy is relevant to a learner’s current writing. This study compared a personalized GenAI coaching package with template-based adaptive metacognitive prompting (AMP) in a nine-week randomized study of Chinese EFL undergraduates.

**Methods:**

Ninety-two participants completed pretest, posttest, and three-week delayed posttest assessments. Both conditions used the same Doubao LLM, interface, and trigger infrastructure. The AMP-Agent condition combined an essay-specific observation, personalized rationale, and an open-ended question; the AMP condition used template prompts.

**Results:**

Analyses of questionnaire item responses found Group × Time interactions for self-regulated learning (SRL; *p* = 0.019) and GenAI dependency (*p* = 0.010), which also survived a retrospective Holm sensitivity correction across the three total outcomes. The SRL total contrasts at posttest and delayed posttest did not survive Bonferroni correction across time points. Self-reported GenAI dependency questionnaire scores were lower in the AMP-Agent group at both later assessments after this correction. Evidence for different writing-score trajectories was insufficient (interaction *p* = 0.101). None of five highlighted behavioral transitions survived correction across 20 transition comparisons.

**Discussion:**

The findings support a difference in self-reported regulation and dependency trajectories between the two prompting packages, while leaving the contribution of rationale alone, sustained writing benefits, and psychological mechanisms unresolved.

## 1 Introduction

Generative AI (GenAI) tools are now embedded in adaptive writing environments for second language (L2) instruction, where they support drafting and revision in real time (Aksakallı and Daşer, 2025; Liu and Liang, 2026). Several recent studies have reported gains in writing quality and learner engagement (Cui et al., 2026; Teng et al., 2026; Zhang et al., 2025). These benefits coexist with concerns that reliance on GenAI may displace learners’ own planning, monitoring, and evaluation (Jia et al., 2026; Zhai et al., 2024). Context-aware scaffolding addresses this concern by detecting opportunities for metacognitive support and issuing relevant prompts during writing (Munshi et al., 2023). A further question is whether learners maintain regulation when that support is no longer available. Scaffolding theory distinguishes contingent support, its gradual withdrawal (fading), and the transfer of responsibility to the learner (van de Pol et al., 2010; Wood et al., 1976). This distinction motivates assessing outcomes after support withdrawal, while recognizing that later outcome scores alone cannot demonstrate strategy internalization.

A relevant design question is whether a personalized coaching package yields different learning trajectories from an active template-based scaffold. Immediate and delayed posttests can assess outcomes after the intervention and their subsequent course; a delayed assessment does not itself establish internalization. Active-control comparisons can hold the delivery infrastructure constant while testing differences in prompt content. The present study uses this approach and examines self-reported SRL, self-reported GenAI dependency, and unaided writing performance as distinct outcomes.

Personalized rationale means an explanation of why a metacognitive strategy is useful for the essay the learner is currently writing. For example, a template may ask a learner to review a thesis statement, whereas a personalized explanation connects that action to a specific difficulty in the draft. Self-Determination Theory (SDT) provides a theoretical reason to examine rationale provision as an autonomy-supportive design feature (Deci et al., 1994; Ryan and Deci, 2020). Whether learners experience such support and internalize the strategy is an empirical question; neither process follows automatically from presenting a rationale.

The present AMP system implemented personalized coaching through a constrained LLM response generator. AMP-Agent denotes the experimental condition; its coaching component generates responses without autonomous planning or tool use. The component generated a situational observation, personalized rationale, and an open-ended question. The AMP control delivered pre-written prompts through the same interface and trigger infrastructure. Because multiple response features varied together, the contrast evaluates the complete coaching package and cannot isolate the effect of rationale. The study followed 92 Chinese EFL undergraduates over 9 weeks.

Hence, the following research questions guided the study:

*RQ1:* Do AMP-Agent and AMP differ in their effects on EFL students’ SRL and GenAI dependency from pretest to delayed posttest?

*RQ2:* Do AMP-Agent and AMP differ in their effects on EFL students’ L2 writing performance from pretest to delayed posttest?

*RQ3:* Do the AMP-Agent and AMP groups show different behavioral transition patterns during GenAI-assisted writing tasks?

## 2 Literature review

### 2.1 Self-regulated learning in GenAI-assisted writing

Self-regulated learning refers to the cyclical process by which learners set goals, monitor their performance, and evaluate their outcomes (Zimmerman, 2000). In technology-supported environments, SRL has taken on added importance as digital tools change the relationship between learner effort and task performance. In L2 writing, SRL is central to the development of academic writing ability among EFL students (Shen and Bai, 2025). SRL strategies are the goal-directed processes through which learners regulate their cognitive, metacognitive, affective, and social engagement with learning (Boulahmel et al., 2025; Greene et al., 2018).

Findings in GenAI-assisted writing are still mixed. On one hand, GenAI tools can serve as metacognitive partners that prompt learners to plan, revise, and reflect (Teng et al., 2026; Yang et al., 2025; Zhang et al., 2025). On the other hand, the availability of high-quality GenAI-generated text may reduce the perceived need for self-regulation. If GenAI can produce acceptable output with minimal learner effort, the incentive to engage in planning or self-evaluation diminishes (Jia et al., 2026). Cheng and Cheung (2026) found that university students interacting with GenAI did not follow the sequential SRL stages proposed in the classic Zimmerman model. GenAI-assisted writing therefore poses a dual challenge for SRL: the same tools that can support metacognitive engagement may also undermine it. This tension calls for scaffolding systems that actively promote metacognitive engagement rather than assuming it will occur on its own.

The six SRL dimensions used here follow Ji et al. (2025): Goal Setting, Task Strategy, Time Management, Self-Evaluation, Environment Structuring, and Help Seeking. They cover how learners organize goals, strategies, time, evaluation, learning conditions, and access to support when using GenAI. These dimensions may respond differently to a writing intervention. Their analyses are therefore reported alongside the total score, with multiplicity sensitivity checks, rather than treating a change in one dimension as evidence for all aspects of self-regulation.

### 2.2 GenAI dependency as a learning risk

Concerns about GenAI dependency arise when reliance on external suggestions displaces learners’ own writing strategies (Zhang et al., 2024; Zhong et al., 2024). The term nevertheless encompasses different constructs across studies, including cognitive, behavioral, functional, and emotional aspects (Wu et al., 2026; Yu and Yan, 2026). The present study operationalizes dependency through Yu and Yan’s (2026) self-report questionnaire. Its content extends beyond potentially problematic reliance to perceived utility and emotional engagement. Accordingly, changes in its scores describe reported tendencies toward GenAI use; they do not by themselves establish excessive reliance, healthier use, or independent writing ability.

The measurement framework in this study is anchored specifically in Yu and Yan’s (2026) three-factor questionnaire. Hou et al. (2026), Wu et al. (2026), and Zhong et al. (2024) provide related accounts of reliance and dependency, but their constructs and instruments are not assumed to be interchangeable. Accordingly, the conceptual labels used in the present study follow the three factors of the instrument actually administered, rather than assuming direct correspondence across distinct dependency frameworks in the prior literature. This distinction separates the wider rationale for studying dependency from the instrument used to operationalize it.

Yu and Yan (2026), Table 8, p. 89 assign Q1–Q6 to Cognitive-Behavioral dependency, Q7–Q12 to Functional dependency, and Q13–Q17 to Emotional dependency. The first factor concerns prioritizing GenAI, reliance on it as a central tool, and displacement of independent strategies. Functional dependency includes perceived usefulness, efficiency, and task support; it should not be equated solely with inability to complete a task without AI. Emotional dependency concerns disclosure, perceived emotional understanding, satisfaction, and mood regulation. These are the three dimensions analyzed here. Supplementary Table S8 maps the published factors to the dataset columns for analysis in this L2 writing study. The unchanged items concern GenAI use generally, so the scores are not specific measures of dependency during writing. The instrument measures self-reported tendencies, not a clinical diagnosis of addiction.

### 2.3 Self-determination theory: autonomy, competence, and relatedness in AMP-based support

SDT proposes that autonomy, competence, and relatedness can support internalization of externally prompted behavior (Ryan and Deci, 2020). These needs informed the intervention design. The asynchronous schedule was intended to offer choice over when to work; the writing tasks and prompts were intended to support competence; and the coaching language was intended to convey a collaborative stance. These are design intentions. The study did not directly assess perceived need satisfaction, expectancy, or internalization, so the outcome measures cannot establish that these psychological processes occurred.

The three response features were designed to connect a writing situation to a learner’s next decision. A situational observation identifies an issue in the current draft; a personalized rationale explains why a metacognitive strategy is relevant to that issue; and an open-ended question invites the learner to decide how to proceed or reflect on the strategy. SDT provides a rationale for making the purpose of an action understandable, but these intended functions are not evidence that each feature produced its proposed effect. Because the features were delivered together, the study evaluates the complete coaching package. Perceived responsiveness, novelty, and expectancy remain alternative explanations for differences between conditions.

### 2.4 Adaptive scaffolding and personalized GenAI coaching

Scaffolding provides support that is responsive to the learner’s current task and competence (Wood et al., 1976). In their review, van de Pol et al. (2010) distinguish contingency, fading, and transfer of responsibility as defining features. Fading refers to the gradual withdrawal of support, whereas maintenance concerns what learners can sustain once support is unavailable. The present study compares prompting packages and assesses outcomes after the intervention; it does not manipulate the rate of fading or directly observe the transfer of regulatory responsibility.

Adaptive scaffolding can combine rules for detecting opportunities for support with a library of response templates. Such templates can respond to a trigger category without explicitly referring to the learner’s essay. Prior work has examined adaptive and planned metacognitive support in GenAI-assisted tasks (J. Liu et al., 2026). The present comparison asks whether replacing templates with a personalized response package changes outcomes when the trigger infrastructure is shared.

The coaching component used here produces a constrained response from the current essay and trigger context. It does not independently plan a sequence of actions, invoke external tools, or select goals. Its educational role is to invite reflection; its computational implementation is dynamic prompt generation. This terminology distinguishes the intervention from stronger forms of autonomous agency. The three-week delayed assessment tests whether outcome differences remain observable after intervention, while the active control narrows the comparison to response-package differences.

## 3 Method

### 3.1 Participants and design

Participants were Chinese EFL undergraduates recruited from a single university through voluntary sign-up across multiple majors. One hundred students enrolled and 92 completed the full nine-week experiment (47 in the AMP-Agent group, 45 in the AMP group; 8% attrition). The study used a randomized two-group pretest–posttest–delayed-posttest design. After the pretest writing assessment at T1 (Week 1), participants were individually randomized by the platform to either the AMP-Agent group or the AMP group through a computer-generated allocation sequence. The posttest and delayed posttest writing assessments were completed at Week 6 (T2) and Week 9 (T3). All writing tasks, including training tasks and assessments, were completed asynchronously. Participants accessed the platform independently within a seven-day window each week, without scheduled class sessions or researcher oversight. This design matched the conditions under which learners use GenAI writing tools in practice. All participants had passed the CET-4 examination with a minimum score of 425, and CET-4 scores did not differ between the two groups, *t*(90) = 0.255, *p* = 0.799, *d* = 0.053.

Both groups used the same AMP system built on the Doubao LLM, with the same user interface and domain knowledge. The independent variable was the response package delivered when the system detected an opportunity for metacognitive prompting: personalized coaching comprising an observation, rationale, and open-ended question (AMP-Agent), or a pre-written prompt (AMP). During Weeks 2–5, participants completed four GenAI-assisted argumentative writing tasks, one per week. At T1, T2, and T3, participants completed independent writing assessments without GenAI assistance. Tasks were adapted from Cambridge IELTS 15/16 Academic Writing Task 2 prompts and used the study’s 300-word, 60-min protocol; scores were not interpreted as official IELTS band scores. The study was approved by the institutional ethics board (SIASSFL260207). All participants provided informed consent before participation.

### 3.2 The AMP system and personalized coaching intervention

The AMP system was developed based on Zimmerman’s (2000) self-regulation model. Both groups shared the same adaptive detection engine, consisting of four behavioral rules (B1 through B4; e.g., word count stagnation, prolonged absence of GenAI interaction), three content rules (C1 through C3; e.g., weak claim detection, off-topic drift), and fixed phase triggers at the planning and reflection stages. When a trigger fired, the system delivered a metacognitive prompt. A trigger budget limited interventions to a maximum of two trigger sessions per writing task and three agent turns per session.

In the AMP group, triggered prompts were drawn from a pre-written template library. These templates directed learners toward metacognitive strategies (e.g., reviewing their thesis statement, checking paragraph coherence) but did not explain why the strategy was relevant to the learner’s specific writing situation or include an open-ended question asking the learner how to proceed within the prompt itself. After each prompt, students responded to a reflection question: “Do you think this suggestion is helpful for your current writing? Why?”

In the AMP-Agent condition, each trigger activated a structured LLM call using the current essay, interaction history, and trigger context. The response contained (a) a non-evaluative situational observation, (b) a brief explanation of why a metacognitive strategy was relevant to the current draft, and (c) an open-ended question inviting reflection. This single-response generator was bounded by the trigger schedule and validation rules; no autonomous planning, external tool use, or multi-step agent workflow is claimed. Figure 1 illustrates the interface.

**Figure 1:**
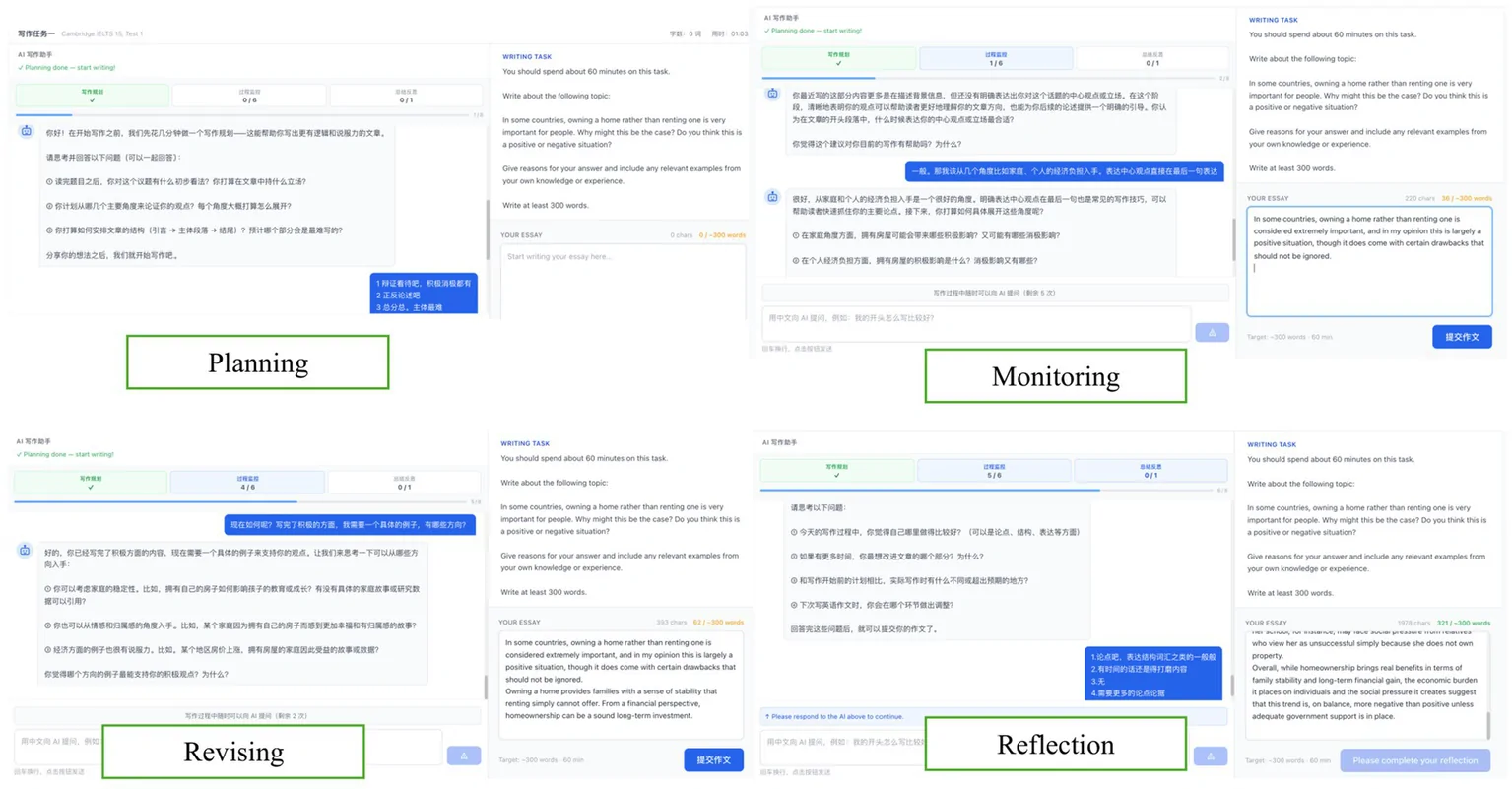
Example of the AMP-Agent interface for L2 writing.

Generated responses passed through a constraint-validation gate checking length (60–120 characters), forbidden direct-correction keywords, and verbatim matching of any quoted essay text. Responses failing a check were replaced with the corresponding AMP template. The shared trigger infrastructure controlled the opportunities to deliver support, but did not match the informational richness of the two packages or the way an open-ended learner question was integrated into the coaching response. The AMP control also included the post-prompt helpfulness reflection described above; this did not make its directive prompt equivalent to the integrated coaching response. Novelty and expectancy remain plausible alternative explanations because they were not independently assessed.

### 3.3 Instruments

SRL was assessed using the 21-item scale for human–machine collaborative environments developed by Ji et al. (2025): Goal Setting (3 items), Task Strategy (4), Time Management (4), Self-Evaluation (4), Environment Structuring (3), and Help Seeking (3). GenAI dependency used the 17-item questionnaire developed by Yu and Yan (2026): Cognitive-Behavioral (6 items), Functional (6), and Emotional (5). The questionnaires were administered without changes to item wording. Responses used five-point scales, with no reverse scoring. Every subscale and total was calculated directly from the item responses. Subscale scores are item means; scale totals are means across all 21 or 17 items, respectively, so all items have equal weight. The dependency mean is the published summed score divided by 17, which changes units but not the test statistics. In the current sample, total-score Cronbach’s α at T1/T2/T3 was 0.903/0.891/0.910 for SRL and 0.783/0.806/0.843 for dependency. Subscale α values ranged from 0.541 to 0.738 (Supplementary Table S9), warranting caution with dimension-specific interpretations. Supplementary Table S8 documents factor-to-column correspondence. Reliability does not establish factor structure or longitudinal measurement invariance in this sample.

Writing was assessed with the ESL Composition Profile (Jacobs, 1981): Content (13–30), Organization (7–20), Vocabulary (7–20), Language Use (5–25), and Mechanics (2–5), summed to a total of 34–100. IELTS prompts supplied argumentative tasks; the Jacobs rubric supplied the analytic outcome definition. Jacobs was selected because the study operationalized writing performance as a multidimensional analytic outcome comprising Content, Organization, Vocabulary, Language Use, and Mechanics, rather than as IELTS proficiency. This pairing allows separate description of content, organization, vocabulary, language use, and mechanics within the study, but does not support conversion to IELTS bands. IELTS Task 2 instead uses Task Response, Coherence and Cohesion, Lexical Resource, and Grammatical Range and Accuracy; spelling and punctuation are distributed within those descriptors rather than forming a separate Mechanics scale (IELTS, 2023). Mechanics therefore has a rubric-specific meaning here. Two trained raters independently scored each essay, with a third rater resolving total-score discrepancies greater than 5 points. Inter-rater reliability, assessed using a two-way random-effects average-measures ICC, was 0.89 for the total and 0.82–0.91 for dimensions.

### 3.4 Experimental procedure

The nine-week experiment proceeded in three phases. Week 1 was the T1 pretest. Each participant wrote an independent argumentative essay in 60 min without GenAI assistance and then completed the SRL and GenAI Dependency questionnaires.

Weeks 2 to 5 formed the training phase. Participants completed one GenAI-assisted writing task per week, each using a new IELTS Academic Writing Task 2 description. Within a seven-day window each week, participants drafted an argumentative essay with LLM access and received metacognitive prompts when AMP triggers fired. After each prompted interaction, participants answered the post-prompt reflection question.

Week 6 was the T2 immediate posttest and Week 9 was the T3 delayed posttest. Both followed the T1 protocol with a new IELTS task, no GenAI access, and full re-administration of the SRL and GenAI Dependency questionnaires. The three-week gap between T2 and T3 allowed assessment of whether group differences in SRL and dependency persisted beyond the immediate posttest (see Figure 2).

**Figure 2:**
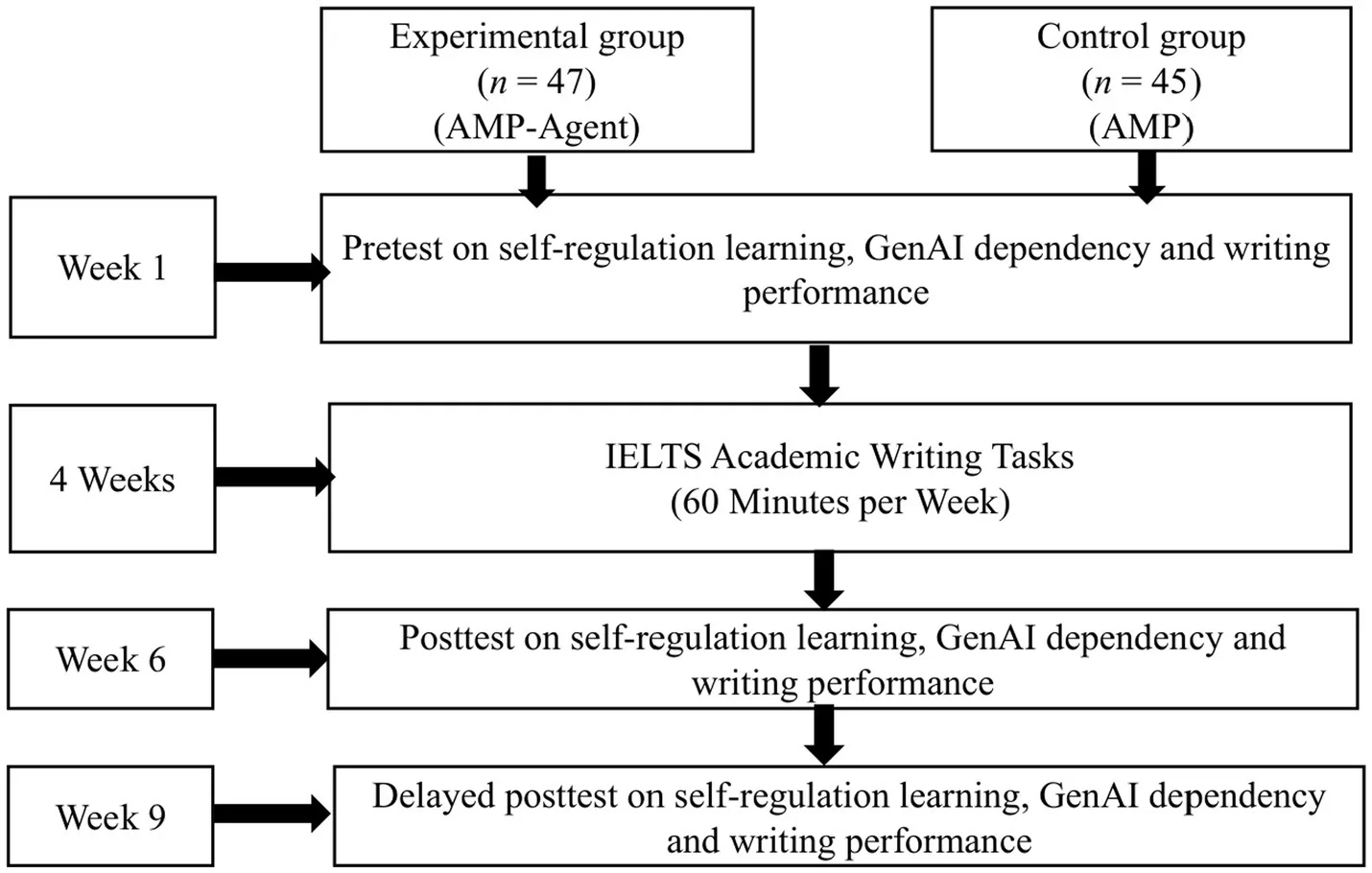
Experimental procedure.

### 3.5 Data analysis

RQ1 and RQ2 were analyzed in R 4.4.1. The analysis file contained 92 completers (47 AMP-Agent, 45 AMP), each observed at T1, T2, and T3, with no missing values in the analyzed outcomes. Participants were the independent units and time was a repeated factor. Each of 17 outcomes was fitted with a 2 × 3 mixed ANOVA using ezANOVA in ez 4.5.0, Type III sums of squares, and sum contrasts. Group × Time was the primary test of differential trajectories. Mauchly tests assessed sphericity; when *p* < 0.05, Greenhouse–Geisser ε was applied to both reported degrees of freedom and *p* values. Partial η^2^ was calculated as effect sum of squares divided by effect plus error sums of squares. Shapiro–Wilk and median-centered Levene tests are supplied as diagnostics, not as rules for removing observations. No additional cases were removed from this complete-case dataset. The eight non-completers are not represented in the analysis, so this is not an intention-to-treat analysis.

Time-specific group contrasts were estimated separately using lmer in lme4 2.0.1: score ~ Group * Time + (1 | participant), fitted by restricted maximum likelihood with a participant random intercept and a common residual variance. Emmeans 2.0.2 used Kenward–Roger degrees of freedom (pbkrtest 0.5.5). The omnibus interaction tests whether group differences vary across assessments; the time-specific contrasts describe group differences at individual assessments. These contrasts come from a separate model with a random-intercept covariance structure, rather than a decomposition of the repeated-measures ANOVA. They are exploratory follow-ups and cannot establish differential trajectories when the primary interaction is not significant. Bonferroni adjustment was explicitly applied across all three group contrasts within each outcome: adjusted *p* = min(3 × unadjusted *p*, 1). Corresponding familywise 95% confidence intervals are given in Tables 1, 2, and unadjusted *p* values in Supplementary Table S7. Cohen’s *d* is the group difference divided by the observed pooled within-time standard deviation. Within-group time contrasts use Bonferroni correction across three time pairs within each group and outcome (Supplementary Tables S4a–b). All tests are two-sided. Dimension-level findings are exploratory. Retrospective Holm sensitivity analyses cover the three total-outcome interactions and, separately, the six SRL, three dependency, and five writing dimension interactions (Supplementary Table S6); these families are not presented as preregistered.

**Table 1:** Between-group simple effects for SRL and GenAI dependency by time point.

Outcome	Time	Δ	95% CI^1^	*p* adj.	*d*
SRL total	T1	−0.02	−0.26, 0.23	1.000	−0.03
SRL total	T2	0.22	−0.03, 0.46	0.108	0.47
SRL total	T3	0.21	−0.04, 0.45	0.136	0.42
SRL–goal setting	T1	0.02	−0.30, 0.33	1.000	0.02
SRL–goal setting	T2	0.20	−0.11, 0.52	0.361	0.35
SRL–goal setting	T3	0.17	−0.14, 0.49	0.579	0.27
SRL–task strategy	T1	0.06	−0.24, 0.36	1.000	0.09
SRL–task strategy	T2	0.27	−0.03, 0.57	0.098	0.50
SRL–Task Strategy	T3	0.27	−0.03, 0.57	0.091	0.45
SRL–Time Mgmt	T1	0.06	−0.26, 0.37	1.000	0.09
SRL–Time Mgmt	T2	0.23	−0.08, 0.55	0.220	0.39
SRL–Time Mgmt	T3	0.05	−0.26, 0.37	1.000	0.08
SRL–Self Eval	T1	0.05	−0.25, 0.35	1.000	0.08
SRL–Self Eval	T2	0.15	−0.15, 0.44	0.703	0.26
SRL–Self Eval	T3	0.10	−0.20, 0.40	1.000	0.18
SRL–Env Structuring	T1	−0.14	−0.46, 0.18	0.892	−0.21
SRL–Env Structuring	T2	0.25	−0.08, 0.57	0.196	0.41
SRL–Env Structuring	T3	0.35	0.03, 0.68	0.027	0.54
SRL–help seeking	T1	−0.21	−0.53, 0.11	0.328	−0.36
SRL–help seeking	T2	0.20	−0.12, 0.51	0.408	0.30
SRL–help seeking	T3	0.36	0.04, 0.67	0.021	0.55
DEP Total	T1	−0.08	−0.21, 0.04	0.348	−0.35
DEP total	T2	−0.23	−0.36, −0.10	<0.001	−0.95
DEP total	T3	−0.22	−0.35, −0.09	<0.001	−0.78
DEP–cognitive-behavioral	T1	−0.06	−0.23, 0.10	1.000	−0.21
DEP–cognitive-behavioral	T2	−0.20	−0.36, −0.03	0.012	−0.65
DEP–cognitive-behavioral	T3	−0.22	−0.39, −0.06	0.003	−0.63
DEP–functional use	T1	−0.10	−0.27, 0.07	0.428	−0.31
DEP–functional use	T2	−0.19	−0.36, −0.02	0.018	−0.58
DEP–functional use	T3	−0.23	−0.40, −0.06	0.003	−0.67
DEP–emotional	T1	−0.09	−0.25, 0.08	0.613	−0.27
DEP–emotional	T2	−0.31	−0.48, −0.15	<0.001	−1.02
DEP–emotional	T3	−0.20	−0.37, −0.04	0.009	−0.59

Δ, AMP-Agent minus AMP; ^1^ familywise 95% confidence intervals and p values use Bonferroni correction across three time points within each outcome. *d* uses the observed pooled standard deviation. Follow-up and dimension-level comparisons are exploratory; full t statistics and Kenward–Roger df are in Supplementary Table S7.

**Table 2:** Between-group simple effects on L2 writing performance by time point.

Outcome	Time	Δ	95% CI^1^	*p* adj.	*d*
Writing performance (total)	T1	0.88	−2.02, 3.79	1.000	0.15
Writing performance (total)	T2	3.18	0.28, 6.08	0.027	0.60
Writing performance (total)	T3	2.41	−0.49, 5.32	0.137	0.40
Writing–content	T1	0.18	−0.71, 1.07	1.000	0.12
Writing–content	T2	0.57	−0.33, 1.46	0.379	0.29
Writing–content	T3	0.64	−0.26, 1.53	0.260	0.35
Writing–organization	T1	0.21	−0.44, 0.86	1.000	0.17
Writing–organization	T2	0.75	0.11, 1.40	0.016	0.56
Writing–organization	T3	0.48	−0.17, 1.12	0.231	0.36
Writing–vocabulary	T1	0.22	−0.45, 0.89	1.000	0.15
Writing–vocabulary	T2	0.62	−0.04, 1.29	0.075	0.49
Writing–vocabulary	T3	0.43	−0.24, 1.10	0.359	0.34
Writing–language use	T1	0.20	−0.56, 0.96	1.000	0.14
Writing–language use	T2	1.00	0.24, 1.76	0.005	0.65
Writing–language use	T3	0.68	−0.08, 1.44	0.093	0.45
Writing–mechanics	T1	0.08	−0.10, 0.27	0.882	0.22
Writing–mechanics	T2	0.23	0.05, 0.42	0.008	0.64
Writing–mechanics	T3	0.19	0.00, 0.37	0.047	0.50

Δ = AMP-Agent minus AMP; ^1^ familywise 95% confidence intervals and p values use Bonferroni correction across three time points within each outcome. *d* uses the observed pooled standard deviation. Follow-up and dimension-level comparisons are exploratory; full t statistics and Kenward–Roger df are in Supplementary Table S7.

For RQ3, first-order transitions were reconstructed within each participant’s writing task, never across task boundaries. Codes were PLAN (planning), MON (monitoring), REV (revision), PASS (passive GenAI use), and REFL (reflection). Within-group adjusted residuals are descriptive (Supplementary Table S5). Between-group conditional probabilities were compared in a 4-origin × 5-destination table. Pearson chi-square homogeneity tests were computed for each originating row and summed for an aggregate omnibus statistic; row *p* values were adjusted across four rows. Two-proportion *z* tests were reported for all 20 cells with Bonferroni correction (adjusted *p* = min(20 × *p*, 1), equivalent to unadjusted α = 0.0025). The five previously highlighted transitions were selected following residual inspection and are not treated as confirmatory hypotheses. These aggregate tests do not model repeated transitions within participants; together with sparse cells, this limits inference and makes the LSA exploratory.

## 4 Results

All 92 completers contributed three assessments. Baseline tests did not detect group differences across the 17 outcomes (all *p* ≥ 0.091); this absence of statistical significance does not establish equivalence. Descriptive statistics are given in Supplementary Tables S1–S3. Questionnaire scores were calculated from item responses as specified in Section 3.3, and writing performance was evaluated using the Jacobs rubric.

### 4.1 Mixed ANOVA findings on self-regulated learning and GenAI dependency (RQ1)

The SRL total Group × Time interaction was *F*(1.74, 156.46) = 4.32, *p* = 0.019, partial η^2^ = 0.046. Group means at T1, T2, and T3 were 3.14, 3.56, 3.83 for AMP-Agent and 3.15, 3.34, 3.62 for AMP. For dependency, the interaction was *F*(2.00, 180.00) = 4.73, *p* = 0.010, partial η^2^ = 0.050; corresponding means were 3.01, 2.67, 2.53 and 3.10, 2.90, 2.75. The interaction *p* values remained below 0.05 after the retrospective Holm correction across three total outcomes (SRL: 0.038; dependency: 0.030). Table 3 reports all total-score ANOVA effects.

**Table 3:** Mixed ANOVA effects for SRL and GenAI dependency.

Outcome	Effect	*F*	*df*	*p*	Partial η^2^
SRL total	Group	2.36	1.00, 90.00	0.128	0.026
	Time	83.78	1.74, 156.46	<0.001	0.482
	Group × Time	4.32	1.74, 156.46	0.019	0.046
DEP total	Group	16.63	1.00, 90.00	<0.001	0.156
	Time	124.22	2.00, 180.00	<0.001	0.580
	Group × Time	4.73	2.00, 180.00	0.010	0.050

Table 1 reports time-specific group contrasts using Bonferroni-adjusted *p* values. The SRL total contrast was positive at T2 (Δ = 0.22, simultaneous 95% CI [−0.03, 0.46], *t*(152.7) = 2.12, adjusted *p* = 0.108, *d* = 0.47) and T3 (Δ = 0.21, simultaneous 95% CI [−0.04, 0.45], *t*(152.7) = 2.02, adjusted *p* = 0.136, *d* = 0.42), but neither survived the three-comparison correction. Thus, the interaction supports a difference in overall trajectories without establishing a corrected total-score advantage at either later time point. Environment Structuring and Help Seeking interactions survived the six-subscale Holm sensitivity analysis (adjusted *p* = 0.014 and 0.003). Their T3 group contrasts survived correction across the three time points within each subscale, but these dimension-level analyses remain exploratory.

Dependency total contrasts favored lower scores in AMP-Agent at T2 (Δ = −0.23, simultaneous 95% CI [−0.36, −0.10], *t*(178.4) = −4.32, adjusted *p* < 0.001, *d* = −0.95) and T3 (Δ = −0.22, simultaneous 95% CI [−0.35, −0.09], *t*(178.4) = −4.12, adjusted *p* < 0.001, *d* = −0.78). Among dimensions, Emotional dependency showed an unadjusted interaction *p* = 0.017, which became 0.052 under the three-dimension Holm sensitivity correction. Cognitive-Behavioral and Functional interactions were not significant. These results do not establish that the intervention affected the emotional dimension more strongly than the other dimensions. All subscale contrasts are reported in Table 1, and within-group comparisons in Supplementary Table S4a.

### 4.2 L2 writing performance (RQ2)

The writing total Group × Time interaction was not significant (*F*(2.00, 180.00) = 2.32, *p* = 0.101, partial η^2^ = 0.025). The evidence is therefore insufficient to conclude that writing-score trajectories differed between the two prompting conditions. Table 4 reports the Group and Time main effects, which do not replace the interaction test.

**Table 4:** Mixed ANOVA effects on L2 writing performance.

Outcome	Effect	*F*	*df*	*p*	Partial η^2^
Writing performance (total)	Group	4.46	1.00, 90.00	0.038	0.047
	Time	17.68	2.00, 180.00	<0.001	0.164
	Group × time	2.32	2.00, 180.00	0.101	0.025

Writing means at T1, T2, and T3 were 70.45, 74.50, 73.88 in AMP-Agent and 69.56, 71.32, 71.46 in AMP. The exploratory T2 group contrast survived correction across time points (Δ = 3.18, simultaneous 95% CI [0.28, 6.08], *t*(159.0) = 2.65, adjusted *p* = 0.027, *d* = 0.60), whereas T3 did not (Δ = 2.41, simultaneous 95% CI [−0.49, 5.32], *t*(159.0) = 2.01, adjusted *p* = 0.137, *d* = 0.40). The Language Use interaction had an unadjusted *p* = 0.039 but Holm-adjusted *p* = 0.197 across five dimensions; no writing dimension interaction survived that sensitivity correction. Table 2 gives all time-specific contrasts, and Supplementary Table S4b gives within-group comparisons. These follow-ups do not demonstrate a sustained differential writing benefit.

### 4.3 Behavioral transition patterns via LSA (RQ3)

Supplementary Table S5 reports all within-group transition counts and adjusted residuals descriptively, without significance stars. The sequence data contained 1,412 within-task transitions from 368 participant-task sequences. The 4 × 5 comparison retained PLAN, MON, REV, and PASS as originating codes and all five destination codes; one REFL-origin transition was outside that comparison, leaving 1,411 analyzed transitions.

The aggregate partitioned chi-square was χ^2^(16) = 31.86, nominal *p* = 0.010. For the four originating-code rows, the PASS row had nominal *p* = 0.008 and Bonferroni-adjusted *p* = 0.032; the other three adjusted row *p* values were not below 0.05 (Supplementary Table S10). These are exploratory aggregate analyses because transitions repeat within participants and tasks, and some expected cell counts are small. Their nominal tests do not provide participant-level evidence of a mechanism.

None of the five highlighted transitions survived Bonferroni correction across the full 20-cell comparison family (Table 5). Adjusted *p*-values were 0.492 for PLAN → REV, 0.933 for PLAN → PASS, 0.782 for MON → PASS, 0.074 for PASS → MON, and 0.165 for PASS → PASS. The observed directions can motivate hypotheses about re-engagement after passive use, but they do not establish reliable individual transition differences. The complete 20-cell results are available in Supplementary Table S10.

**Table 5:** Between-group comparison of key conditional transition probabilities (CTP).

Transition	Agent CTP	AMP CTP	Δ CTP	*z*	*p* raw	*p* adj.
PLAN → REV	0.184	0.105	0.080	2.25	0.025	0.492
PLAN → PASS	0.398	0.497	−0.099	−1.99	0.047	0.933
MON → PASS	0.234	0.329	−0.096	−2.06	0.039	0.782
PASS → MON	0.246	0.143	0.103	2.90	0.004	0.074
PASS → PASS	0.285	0.398	−0.113	−2.64	0.008	0.165

All five highlighted transitions are non-significant after Bonferroni correction across 20 cells. Aggregate transition tests are exploratory and do not account for repeated transitions within participants.

## 5 Discussion

Under shared delivery conditions, the personalized coaching package and template-based prompting yielded different trajectories in self-reported SRL and GenAI dependency. The study thus provides evidence that prompt content can matter for learners’ reported regulation and relationship with GenAI. Evidence for differential improvement in unaided writing was insufficient. Evaluating these outcomes separately is essential: changes in self-reports do not establish gains in independent task performance, and the differing statistical results do not themselves demonstrate a dissociation between the two.

### 5.1 Self-regulation and GenAI dependency

The SRL interaction indicates a difference in change across the three assessments, but the corrected total-score contrasts at T2 and T3 were not significant. The evidence therefore supports different trajectories without establishing an SRL total-score advantage at either later assessment. Environment Structuring and Help Seeking showed notable exploratory dimension-level patterns. The delayed assessment extends observation to three weeks after the intervention, but neither the duration of follow-up nor these self-reports establish that scaffolded strategies became independently maintained skills. Longer follow-up and direct observation of unaided regulation would address that question.

The dependency interaction and corrected later group contrasts support lower self-reported GenAI dependency questionnaire scores in the coaching condition. One possible explanation is that linking an observed writing issue to a strategy rationale and a learner decision encouraged more reflective engagement. This account follows the intended functions of the complete package, rather than identifying rationale as its active ingredient. SDT offers a theoretical basis for examining such explanations (Ryan and Deci, 2020), but perceived autonomy, competence, relatedness, and internalization were not measured. The findings therefore motivate testing these processes directly, alongside alternatives such as novelty and perceived responsiveness.

The three dependency factors should be interpreted according to their measurement content. Functional items partly reflect perceived utility, so lower scores need not mean that learners became more capable of writing without AI. Emotional dependency did not show a robust interaction after correction across dependency dimensions; a claim of uniquely strong affective disengagement is therefore unwarranted. The modest subscale reliabilities further limit finely differentiated claims.

### 5.2 Independent writing performance

Both groups showed higher mean writing scores at later assessments, but the primary interaction did not support differential improvement. The corrected T2 group contrast provides an exploratory description of the posttest gap. The T3 contrast did not survive correction. A difference at one assessment does not establish a difference in change across assessments, and these results do not establish a writing advantage retained after support withdrawal.

Language Use and Mechanics are dimensions of the Jacobs rubric, not direct measures of metacognitive strategy internalization. A possible pathway is that deliberate monitoring increases attention to language form, but this pathway was not tested. The Language Use interaction did not survive correction across writing dimensions, while Mechanics lacked interaction support. These patterns cannot establish automaticity, integrated regulation, or a specific transfer mechanism.

Rubric choice also limits generalization. Mechanics is scored separately in Jacobs, whereas IELTS incorporates relevant surface features within other criteria. The present scores describe analytic writing performance under the study protocol, not changes in IELTS proficiency bands. Replication using another rubric and direct measures of strategy use would help assess whether any writing-related pattern generalizes.

### 5.3 Behavioral dynamics during GenAI-assisted writing

The transition patterns provide descriptive hypotheses about regulation during assisted writing. AMP-Agent had numerically more PASS → MON transitions and fewer PASS → PASS transitions than AMP, but neither survived the stated 20-comparison correction. These patterns do not establish passive lock-in as a behavioral mechanism. The aggregate omnibus test does not identify which individual transition differs reliably.

Repeated transitions within participants and tasks also limit the independence assumptions of aggregate LSA. Future studies could preregister transition hypotheses and use participant-level or multilevel sequence models. The present sequence results should not be used to explain the outcome effects causally or to attribute a behavioral change specifically to rationale provision.

### 5.4 Pedagogical and design implications

Teachers can use the three-component response format as a practical design example: identify a specific feature of a draft, explain why a relevant strategy may help, and ask the learner to decide how to respond. Instructors could ask students to retain a brief record of the strategy chosen and its application, and include unaided writing tasks when evaluating learning. These are pedagogical suggestions rather than separately tested intervention components. The study does not establish that adopting the format alone improves independent writing.

For developers, the comparison illustrates how contextualized coaching can be implemented within an existing trigger-and-template architecture. Evaluation should distinguish learner engagement, self-reported dependency, and independent performance. Future component studies should match response length and interactivity while varying rationale and personalization separately, and measure perceived credibility or expectancy to test alternative explanations.

### 5.5 Limitations and future directions

Several limitations qualify the findings. The sample came from one Chinese university, and analyses include only 92 of the 100 enrolled participants. Attrition and a short three-week follow-up limit generalization and conclusions about durability. SRL and dependency were self-reported, several subscales had modest internal consistency, and longitudinal measurement invariance was not established. The Functional dependency factor also includes favorable evaluations of AI utility, so a decrease is not by itself evidence of reduced maladaptive use.

The intervention varied a response package rather than rationale alone. Personalized observations, open-ended questioning, response richness, novelty, and expectancy could contribute to the differences; their effects cannot be separated with this design. The study did not directly measure psychological need satisfaction or internalization. Writing findings are limited by the non-significant total interaction and rubric-specific dimensions. Aggregate LSA does not account for participant-level dependence and should be treated as exploratory. Replication with longer follow-up, other LLMs and populations, and a factorial component design is needed.

## 6 Conclusion

In this active-control study, the personalized coaching package and template-based prompting produced different trajectories in self-reported SRL and GenAI dependency questionnaire scores. Corrected group contrasts supported lower dependency scores at posttest and delayed posttest, but did not establish corresponding SRL total-score advantages at those time points. Evidence for different unaided writing trajectories was insufficient, and no highlighted LSA transition survived the stated correction.

The study identifies differences in learners’ reported regulation and GenAI dependency under two forms of adaptive prompting delivered through a shared infrastructure. It also shows why these self-reports must be considered alongside independent performance when evaluating coaching. Personalized rationale was delivered with situational observations and open-ended questions; its separate contribution and the proposed internalization process remain questions for component-level studies with direct psychological and behavioral measures.

## Data Availability

The raw data supporting the conclusions of this article will be made available by the authors, without undue reservation.

## References

[ref1] Aksakallı C. Daşer Z. (2025). Unlocking EFL learners’ insights into ChatGPT use for L2 writing: the impacts of usage frequency and gender variations. Curr. Psychol. 44, 7957–7977. doi: 10.1007/s12144-025-07437-3

[ref4] Boulahmel A. Djelil F. Smits G. (2025). Investigating self-regulated learning measurement based on trace data: a systematic literature review. Technol. Knowl. Learn. 30, 119–156. doi: 10.1007/s10758-025-09816-y

[ref5] Cheng M. Cheung K. K. C. (2026). University students’ sequential self-regulatory learning actions for completing cognitive-demanding and cognitive-less-demanding tasks with GenAI: ordered network analysis. Interact. Learn. Environ., 1–16. doi: 10.1080/10494820.2025.2609793

[ref6] Cui H. Mahfoodh O. H. A. Dong H. Ulanbek M. (2026). An assessment framework for human-AI collaborative competence in second language writing: evidence from the Chinese EFL context. System 137:103937. doi: 10.1016/j.system.2025.103937

[ref8] Deci E. L. Eghrari H. Patrick B. C. Leone D. R. (1994). Facilitating internalization: the self-determination theory perspective. J. Pers. 62, 119–142. doi: 10.1111/j.1467-6494.1994.tb00797.x, 8169757

[ref9] Greene J. A. Copeland D. Z. Deekens V. M. Freed R. (2018). “Self-regulated learning processes and multiple source use in and out of school,” in Handbook of Multiple Source Use, Eds. J. L. G. Braasch, I. Bråten, and M. T. McCrudden. New York, NY: (Routledge), 320–338.

[ref10] Hou C. Zhu G. Liu Y. Sudarshan V. Chong J. L. L. Zhang F. Y. . (2026). The effects of critical thinking intervention on reliance behaviors, problem-solving quality, and creativity during human-generative AI collaborative learning. Comput. Educ. 247:105576. doi: 10.1016/j.compedu.2026.105576

[ref11] IELTS (2023). Writing band descriptors. Updated May 2023. Available online at: https://ielts.org/cdn/ielts-guides/ielts-writing-band-descriptors.pdf (Accessed October 8, 2026).

[ref12] Jacobs H. L. (1981). Testing ESL Composition: A Practical Approach. English Composition Program. Rowley, MA: ERIC.

[ref13] Ji Y. Zhong M.-X. Lyu S.-Y. Li T.-D. Zhan Z.-H. (2025). Development and application of self-regulated learning ability assessment scale for human-machine collaborative environments. Mod. Educ. Technol. 35:35. doi: 10.3969/j.issn.1009-8097.2025.09.004

[ref14] Jia C. Jin Z. Lu Y. (2026). Exploring learners’ self-regulated learning behaviors in generative AI-assisted academic writing: a cluster and sequential analysis. Interact. Learn. Environ. 34, 1–21. doi: 10.1080/10494820.2026.2658206

[ref17] Liu C. Liang M. (2026). The effects of AI-assisted feedback on students’ perceptions, emotions, and learning outcomes in L2 writing: a three-level meta-analysis. System 139: 104018 doi: 10.1016/j.system.2026.104018

[ref18] Liu J. Zhang Y. Li W. Wang Q. Niu P. Zhang X. (2026). Adaptive vs. planned metacognitive scaffolding for computational thinking: evidence from generative AI-supported programming in elementary education. Comput. Educ. 241:105473. doi: 10.1016/j.compedu.2025.105473

[ref19] Munshi A. Biswas G. Baker R. Ocumpaugh J. Hutt S. Paquette L. (2023). Analysing adaptive scaffolds that help students develop self-regulated learning behaviours. J. Comput. Assist. Learn. 39, 351–368. doi: 10.1111/jcal.12761

[ref21] Ryan R. M. Deci E. L. (2020). Intrinsic and extrinsic motivation from a self-determination theory perspective: definitions, theory, practices, and future directions. Contemp. Educ. Psychol. 61:101860. doi: 10.1016/j.cedpsych.2020.101860

[ref22] Shen B. Bai B. (2025). Explorations into EFL learners’ self-regulated writing strategy use in writing for scholarly publications: a case study of Chinese PhD students. Lang. Aware. 1–22. doi: 10.1080/09658416.2025.2585007

[ref23] Teng L. S. Deng X. Yang J. (2026). Can GenAI-empowered feedback promote L2 learners’ self-regulation strategic behavior and writing performance? System 138:103970. doi: 10.1016/j.system.2026.103970

[ref24] van de Pol J. Volman M. Beishuizen J. (2010). Scaffolding in teacher–student interaction: a decade of research. Educ. Psychol. Rev. 22, 271–296. doi: 10.1007/s10648-010-9127-6, 30311153

[ref26] Wood D. Bruner J. S. Ross G. (1976). The role of tutoring in problem solving. J. Child Psychol. Psychiatry 17, 89–100. doi: 10.1111/j.1469-7610.1976.tb00381.x, 932126

[ref27] Wu H. Ni H. Luo W. Wu T. (2026). Development and validation of the AI dependence scale for Chinese undergraduates and a preliminary exploration. Front. Psychol. 16:1725393. doi: 10.3389/fpsyg.2025.1725393, 41635770 PMC12862064

[ref29] Yang C. Li R. Yang L. (2025). Revisiting trends in GenAI-assisted second language writing: retrospect and prospect. J. Educ. Comput. Res. 63, 1819–1863. doi: 10.1177/07356331251367309

[ref30] Yu Z. Yan J. (2026). Development and network analysis validation of the generative artificial intelligence dependency questionnaire for students. J. Distance Educ. 44, 83–91. doi: 10.15881/j.cnki.cn33-1304/g4.2026.01.009

[ref31] Zhai C. Wibowo S. Li L. D. (2024). The effects of over-reliance on AI dialogue systems on students’ cognitive abilities: a systematic review. Smart Learn. Environ. 11:28. doi: 10.1186/s40561-024-00316-7

[ref33] Zhang Z. Aubrey S. Huang X. Chiu T. K. F. (2025). The role of generative AI and hybrid feedback in improving L2 writing skills: a comparative study. Innov. Lang. Learn. Teach.writing strategy use in writing for scholarly publications, 1–19. doi: 10.1080/17501229.2025.2503890, 37339054

[ref34] Zhang S. Zhao X. Zhou T. Kim J. H. (2024). Do you have AI dependency? The roles of academic self-efficacy, academic stress, and performance expectations on problematic AI usage behavior. Int. J. Educ. Technol. High. Educ. 21:34. doi: 10.1186/s41239-024-00467-0

[ref36] Zhong W. Luo J. Lyu Y. (2024). How do personal attributes shape AI dependency in Chinese higher education context? Insights from needs frustration perspective. PLoS One 19:e0313314. doi: 10.1371/journal.pone.0313314, 39485818 PMC11530054

[ref37] Zimmerman B. J. (2000). Self-efficacy: an essential motive to learn. Contemp. Educ. Psychol. 25, 82–91. doi: 10.1006/ceps.1999.1016, 10620383

