# Supplementary material for: Personalized GenAI coaching and self-regulation in L2 writing across a nine-week active-control study
Source: Front Psychol. 2026 Oct 14;17:1904842. doi: 10.3389/fpsyg.2026.1904842 (PMC13713743; doi:10.3389/fpsyg.2026.1904842)
Supplement: Supplementary file 1 [file Table_1.docx]

Supplementary Materials for the Round 1 Revision

These tables accompany the item-derived reanalysis of 92 completers. T1, T2, and T3 denote pretest, immediate posttest, and delayed posttest. AMP-Agent n = 47; AMP n = 45. Questionnaire totals are item means; writing scores use the Jacobs rubric.

# Table S1 Descriptive statistics for SRL

| **Outcome** | **Agent T1** | **AMP T1** | **Agent T2** | **AMP T2** | **Agent T3** | **AMP T3** |
| --- | --- | --- | --- | --- | --- | --- |
| SRL Total | 3.14 (0.50) | 3.15 (0.54) | 3.56 (0.51) | 3.34 (0.41) | 3.83 (0.51) | 3.62 (0.48) |
| SRL - Goal Setting | 3.15 (0.63) | 3.13 (0.70) | 3.58 (0.59) | 3.38 (0.59) | 3.83 (0.66) | 3.66 (0.58) |
| SRL - Task Strategy | 3.16 (0.63) | 3.10 (0.67) | 3.58 (0.56) | 3.31 (0.51) | 3.88 (0.63) | 3.61 (0.57) |
| SRL - Time Mgmt | 3.18 (0.58) | 3.12 (0.69) | 3.46 (0.66) | 3.23 (0.56) | 3.68 (0.62) | 3.63 (0.64) |
| SRL - Self Eval | 3.23 (0.62) | 3.18 (0.67) | 3.57 (0.57) | 3.43 (0.54) | 3.78 (0.53) | 3.68 (0.60) |
| SRL - Env Structuring | 2.99 (0.70) | 3.13 (0.65) | 3.56 (0.67) | 3.31 (0.52) | 3.85 (0.62) | 3.50 (0.69) |
| SRL - Help Seeking | 3.06 (0.60) | 3.27 (0.57) | 3.60 (0.69) | 3.40 (0.59) | 3.99 (0.59) | 3.64 (0.70) |

Values are mean (SD).

# Table S2 Descriptive statistics for DEP

| **Outcome** | **Agent T1** | **AMP T1** | **Agent T2** | **AMP T2** | **Agent T3** | **AMP T3** |
| --- | --- | --- | --- | --- | --- | --- |
| DEP Total | 3.01 (0.25) | 3.10 (0.24) | 2.67 (0.25) | 2.90 (0.23) | 2.53 (0.31) | 2.75 (0.25) |
| DEP - Cognitive-Behavioral | 3.04 (0.28) | 3.10 (0.33) | 2.67 (0.32) | 2.87 (0.29) | 2.54 (0.41) | 2.76 (0.30) |
| DEP - Functional Use | 3.05 (0.37) | 3.15 (0.28) | 2.75 (0.32) | 2.94 (0.34) | 2.57 (0.35) | 2.80 (0.34) |
| DEP - Emotional | 2.93 (0.29) | 3.02 (0.34) | 2.59 (0.35) | 2.90 (0.26) | 2.48 (0.36) | 2.68 (0.32) |

Values are mean (SD).

# Table S3 Descriptive statistics for Writing

| **Outcome** | **Agent T1** | **AMP T1** | **Agent T2** | **AMP T2** | **Agent T3** | **AMP T3** |
| --- | --- | --- | --- | --- | --- | --- |
| Writing Performance (Total) | 70.45 (5.66) | 69.56 (6.02) | 74.50 (5.62) | 71.32 (4.97) | 73.88 (5.80) | 71.46 (6.34) |
| Writing - Content | 20.05 (1.54) | 19.87 (1.54) | 21.02 (1.97) | 20.46 (1.93) | 20.93 (1.79) | 20.29 (1.81) |
| Writing - Organization | 14.62 (1.19) | 14.41 (1.19) | 15.56 (1.43) | 14.81 (1.26) | 15.31 (1.23) | 14.84 (1.40) |
| Writing - Vocabulary | 14.27 (1.27) | 14.06 (1.56) | 15.08 (1.34) | 14.46 (1.22) | 15.01 (1.20) | 14.58 (1.31) |
| Writing - Language Use | 17.26 (1.46) | 17.07 (1.46) | 18.36 (1.74) | 17.36 (1.28) | 18.17 (1.45) | 17.49 (1.60) |
| Writing - Mechanics | 4.24 (0.36) | 4.16 (0.36) | 4.47 (0.36) | 4.24 (0.37) | 4.46 (0.35) | 4.27 (0.40) |

Values are mean (SD).

# Table S4a Within-group time comparisons

| **Outcome** | **Group** | **Time pair** | **Δ** | **95% CI** | **Bonferroni-adjusted *p*** |
| --- | --- | --- | --- | --- | --- |
| SRL Total | AMP-Agent | T1 - T2 | -0.42 | -0.57, -0.27 | <.001 |
| SRL Total | AMP-Agent | T1 - T3 | -0.69 | -0.84, -0.54 | <.001 |
| SRL Total | AMP-Agent | T2 - T3 | -0.27 | -0.42, -0.12 | <.001 |
| SRL Total | AMP | T1 - T2 | -0.19 | -0.34, -0.03 | .012 |
| SRL Total | AMP | T1 - T3 | -0.47 | -0.62, -0.31 | <.001 |
| SRL Total | AMP | T2 - T3 | -0.28 | -0.43, -0.13 | <.001 |
| SRL - Goal Setting | AMP-Agent | T1 - T2 | -0.43 | -0.69, -0.18 | <.001 |
| SRL - Goal Setting | AMP-Agent | T1 - T3 | -0.68 | -0.94, -0.43 | <.001 |
| SRL - Goal Setting | AMP-Agent | T2 - T3 | -0.25 | -0.50, 0.01 | .059 |
| SRL - Goal Setting | AMP | T1 - T2 | -0.24 | -0.50, 0.02 | .073 |
| SRL - Goal Setting | AMP | T1 - T3 | -0.53 | -0.79, -0.27 | <.001 |
| SRL - Goal Setting | AMP | T2 - T3 | -0.28 | -0.54, -0.02 | .029 |
| SRL - Task Strategy | AMP-Agent | T1 - T2 | -0.42 | -0.64, -0.20 | <.001 |
| SRL - Task Strategy | AMP-Agent | T1 - T3 | -0.72 | -0.94, -0.50 | <.001 |
| SRL - Task Strategy | AMP-Agent | T2 - T3 | -0.30 | -0.52, -0.08 | .003 |
| SRL - Task Strategy | AMP | T1 - T2 | -0.21 | -0.43, 0.01 | .068 |
| SRL - Task Strategy | AMP | T1 - T3 | -0.51 | -0.73, -0.28 | <.001 |
| SRL - Task Strategy | AMP | T2 - T3 | -0.29 | -0.52, -0.07 | .005 |
| SRL - Time Mgmt | AMP-Agent | T1 - T2 | -0.28 | -0.52, -0.04 | .016 |
| SRL - Time Mgmt | AMP-Agent | T1 - T3 | -0.50 | -0.74, -0.26 | <.001 |
| SRL - Time Mgmt | AMP-Agent | T2 - T3 | -0.22 | -0.46, 0.02 | .092 |
| SRL - Time Mgmt | AMP | T1 - T2 | -0.11 | -0.35, 0.14 | .909 |
| SRL - Time Mgmt | AMP | T1 - T3 | -0.51 | -0.75, -0.26 | <.001 |
| SRL - Time Mgmt | AMP | T2 - T3 | -0.40 | -0.65, -0.15 | <.001 |
| SRL - Self Eval | AMP-Agent | T1 - T2 | -0.35 | -0.58, -0.11 | .001 |
| SRL - Self Eval | AMP-Agent | T1 - T3 | -0.55 | -0.78, -0.31 | <.001 |
| SRL - Self Eval | AMP-Agent | T2 - T3 | -0.20 | -0.44, 0.03 | .115 |
| SRL - Self Eval | AMP | T1 - T2 | -0.25 | -0.49, -0.01 | .037 |
| SRL - Self Eval | AMP | T1 - T3 | -0.50 | -0.74, -0.26 | <.001 |
| SRL - Self Eval | AMP | T2 - T3 | -0.25 | -0.49, -0.01 | .037 |
| SRL - Env Structuring | AMP-Agent | T1 - T2 | -0.57 | -0.82, -0.31 | <.001 |
| SRL - Env Structuring | AMP-Agent | T1 - T3 | -0.86 | -1.11, -0.60 | <.001 |
| SRL - Env Structuring | AMP-Agent | T2 - T3 | -0.29 | -0.54, -0.04 | .018 |
| SRL - Env Structuring | AMP | T1 - T2 | -0.18 | -0.44, 0.08 | .296 |
| SRL - Env Structuring | AMP | T1 - T3 | -0.36 | -0.62, -0.10 | .003 |
| SRL - Env Structuring | AMP | T2 - T3 | -0.19 | -0.44, 0.07 | .257 |
| SRL - Help Seeking | AMP-Agent | T1 - T2 | -0.53 | -0.78, -0.28 | <.001 |
| SRL - Help Seeking | AMP-Agent | T1 - T3 | -0.93 | -1.18, -0.68 | <.001 |
| SRL - Help Seeking | AMP-Agent | T2 - T3 | -0.40 | -0.65, -0.15 | <.001 |
| SRL - Help Seeking | AMP | T1 - T2 | -0.13 | -0.38, 0.13 | .696 |
| SRL - Help Seeking | AMP | T1 - T3 | -0.36 | -0.62, -0.11 | .002 |
| SRL - Help Seeking | AMP | T2 - T3 | -0.24 | -0.49, 0.02 | .076 |
| DEP Total | AMP-Agent | T1 - T2 | 0.34 | 0.25, 0.43 | <.001 |
| DEP Total | AMP-Agent | T1 - T3 | 0.48 | 0.39, 0.57 | <.001 |
| DEP Total | AMP-Agent | T2 - T3 | 0.14 | 0.05, 0.23 | <.001 |
| DEP Total | AMP | T1 - T2 | 0.19 | 0.10, 0.28 | <.001 |
| DEP Total | AMP | T1 - T3 | 0.34 | 0.25, 0.44 | <.001 |
| DEP Total | AMP | T2 - T3 | 0.15 | 0.06, 0.24 | <.001 |
| DEP - Cognitive-Behavioral | AMP-Agent | T1 - T2 | 0.37 | 0.24, 0.49 | <.001 |
| DEP - Cognitive-Behavioral | AMP-Agent | T1 - T3 | 0.50 | 0.38, 0.63 | <.001 |
| DEP - Cognitive-Behavioral | AMP-Agent | T2 - T3 | 0.14 | 0.01, 0.26 | .025 |
| DEP - Cognitive-Behavioral | AMP | T1 - T2 | 0.23 | 0.11, 0.36 | <.001 |
| DEP - Cognitive-Behavioral | AMP | T1 - T3 | 0.34 | 0.22, 0.47 | <.001 |
| DEP - Cognitive-Behavioral | AMP | T2 - T3 | 0.11 | -0.02, 0.24 | .113 |
| DEP - Functional Use | AMP-Agent | T1 - T2 | 0.30 | 0.16, 0.44 | <.001 |
| DEP - Functional Use | AMP-Agent | T1 - T3 | 0.48 | 0.33, 0.62 | <.001 |
| DEP - Functional Use | AMP-Agent | T2 - T3 | 0.17 | 0.03, 0.32 | .011 |
| DEP - Functional Use | AMP | T1 - T2 | 0.21 | 0.07, 0.36 | .002 |
| DEP - Functional Use | AMP | T1 - T3 | 0.35 | 0.20, 0.49 | <.001 |
| DEP - Functional Use | AMP | T2 - T3 | 0.14 | -0.01, 0.28 | .072 |
| DEP - Emotional | AMP-Agent | T1 - T2 | 0.34 | 0.21, 0.48 | <.001 |
| DEP - Emotional | AMP-Agent | T1 - T3 | 0.46 | 0.32, 0.59 | <.001 |
| DEP - Emotional | AMP-Agent | T2 - T3 | 0.11 | -0.02, 0.25 | .145 |
| DEP - Emotional | AMP | T1 - T2 | 0.12 | -0.02, 0.25 | .131 |
| DEP - Emotional | AMP | T1 - T3 | 0.34 | 0.20, 0.48 | <.001 |
| DEP - Emotional | AMP | T2 - T3 | 0.22 | 0.08, 0.36 | <.001 |

Differences are first time point minus second. Bonferroni adjustment covers three time pairs within each group and outcome. All comparisons are exploratory follow-ups.

# Table S4b Within-group time comparisons

| **Outcome** | **Group** | **Time pair** | **Δ** | **95% CI** | **Bonferroni-adjusted *p*** |
| --- | --- | --- | --- | --- | --- |
| Writing Performance (Total) | AMP-Agent | T1 - T2 | -4.05 | -5.89, -2.22 | <.001 |
| Writing Performance (Total) | AMP-Agent | T1 - T3 | -3.43 | -5.26, -1.60 | <.001 |
| Writing Performance (Total) | AMP-Agent | T2 - T3 | 0.62 | -1.21, 2.46 | 1.000 |
| Writing Performance (Total) | AMP | T1 - T2 | -1.76 | -3.63, 0.11 | .073 |
| Writing Performance (Total) | AMP | T1 - T3 | -1.90 | -3.77, -0.03 | .046 |
| Writing Performance (Total) | AMP | T2 - T3 | -0.14 | -2.01, 1.73 | 1.000 |
| Writing - Content | AMP-Agent | T1 - T2 | -0.97 | -1.60, -0.35 | <.001 |
| Writing - Content | AMP-Agent | T1 - T3 | -0.88 | -1.50, -0.25 | .002 |
| Writing - Content | AMP-Agent | T2 - T3 | 0.10 | -0.53, 0.72 | 1.000 |
| Writing - Content | AMP | T1 - T2 | -0.59 | -1.22, 0.05 | .082 |
| Writing - Content | AMP | T1 - T3 | -0.42 | -1.06, 0.22 | .338 |
| Writing - Content | AMP | T2 - T3 | 0.17 | -0.47, 0.80 | 1.000 |
| Writing - Organization | AMP-Agent | T1 - T2 | -0.94 | -1.43, -0.46 | <.001 |
| Writing - Organization | AMP-Agent | T1 - T3 | -0.70 | -1.18, -0.21 | .002 |
| Writing - Organization | AMP-Agent | T2 - T3 | 0.25 | -0.24, 0.73 | .650 |
| Writing - Organization | AMP | T1 - T2 | -0.40 | -0.89, 0.10 | .163 |
| Writing - Organization | AMP | T1 - T3 | -0.43 | -0.92, 0.07 | .117 |
| Writing - Organization | AMP | T2 - T3 | -0.03 | -0.52, 0.47 | 1.000 |
| Writing - Vocabulary | AMP-Agent | T1 - T2 | -0.81 | -1.23, -0.38 | <.001 |
| Writing - Vocabulary | AMP-Agent | T1 - T3 | -0.73 | -1.16, -0.31 | <.001 |
| Writing - Vocabulary | AMP-Agent | T2 - T3 | 0.07 | -0.35, 0.50 | 1.000 |
| Writing - Vocabulary | AMP | T1 - T2 | -0.40 | -0.84, 0.03 | .078 |
| Writing - Vocabulary | AMP | T1 - T3 | -0.52 | -0.96, -0.09 | .013 |
| Writing - Vocabulary | AMP | T2 - T3 | -0.12 | -0.55, 0.32 | 1.000 |
| Writing - Language Use | AMP-Agent | T1 - T2 | -1.09 | -1.61, -0.57 | <.001 |
| Writing - Language Use | AMP-Agent | T1 - T3 | -0.91 | -1.43, -0.39 | <.001 |
| Writing - Language Use | AMP-Agent | T2 - T3 | 0.18 | -0.34, 0.70 | 1.000 |
| Writing - Language Use | AMP | T1 - T2 | -0.29 | -0.82, 0.24 | .567 |
| Writing - Language Use | AMP | T1 - T3 | -0.42 | -0.95, 0.11 | .167 |
| Writing - Language Use | AMP | T2 - T3 | -0.13 | -0.66, 0.40 | 1.000 |
| Writing - Mechanics | AMP-Agent | T1 - T2 | -0.23 | -0.37, -0.10 | <.001 |
| Writing - Mechanics | AMP-Agent | T1 - T3 | -0.21 | -0.35, -0.08 | <.001 |
| Writing - Mechanics | AMP-Agent | T2 - T3 | 0.02 | -0.11, 0.15 | 1.000 |
| Writing - Mechanics | AMP | T1 - T2 | -0.08 | -0.22, 0.05 | .420 |
| Writing - Mechanics | AMP | T1 - T3 | -0.11 | -0.24, 0.03 | .153 |
| Writing - Mechanics | AMP | T2 - T3 | -0.03 | -0.16, 0.11 | 1.000 |

Differences are first time point minus second. Bonferroni adjustment covers three time pairs within each group and outcome. All comparisons are exploratory follow-ups.

# Table S5 All descriptive within-group transitions

| **Transition** | **Agent count** | **Agent residual** | **AMP count** | **AMP residual** |
| --- | --- | --- | --- | --- |
| PLAN → PLAN | 7 | 1.05 | 6 | 1.94 |
| PLAN → MON | 74 | 2.56 | 68 | 4.29 |
| PLAN → REV | 38 | 3.31 | 20 | 1.58 |
| PLAN → PASS | 82 | 3.24 | 95 | 3.48 |
| PLAN → REFL | 5 | -8.95 | 2 | -9.51 |
| MON → PLAN | 4 | -0.64 | 3 | 0.23 |
| MON → MON | 66 | 0.69 | 43 | 0.65 |
| MON → REV | 25 | -0.21 | 13 | 0.03 |
| MON → PASS | 50 | -2.84 | 54 | -1.93 |
| MON → REFL | 69 | 2.68 | 51 | 1.41 |
| REV → PLAN | 4 | 1.34 | 1 | 0.15 |
| REV → MON | 18 | -1.95 | 15 | 0.70 |
| REV → REV | 10 | -0.26 | 7 | 1.51 |
| REV → PASS | 31 | 0.85 | 10 | -3.18 |
| REV → REFL | 26 | 0.86 | 20 | 1.86 |
| PASS → PLAN | 3 | -1.33 | 1 | -2.08 |
| PASS → MON | 56 | -1.79 | 38 | -4.91 |
| PASS → REV | 16 | -2.82 | 13 | -2.32 |
| PASS → PASS | 65 | -0.95 | 106 | 0.23 |
| PASS → REFL | 88 | 5.46 | 108 | 6.50 |

Adjusted residuals describe departures from row-by-column independence within each group. No significance stars are used. These are not corrected between-group tests.

# Table S6 Interaction tests and multiplicity sensitivity

| **Outcome** | **F** | ***df*** | **Unadjusted *p*** | **Holm-adjusted *p*** | **Partial η²** |
| --- | --- | --- | --- | --- | --- |
| SRL Total | 4.32 | 1.74, 156.46 | .019 | .038 | 0.046 |
| SRL - Goal Setting | 0.89 | 1.83, 164.75 | .405 | 1.000 | 0.010 |
| SRL - Task Strategy | 1.79 | 2.00, 180.00 | .169 | .677 | 0.020 |
| SRL - Time Mgmt | 1.05 | 2.00, 180.00 | .353 | 1.000 | 0.011 |
| SRL - Self Eval | 0.24 | 2.00, 180.00 | .788 | 1.000 | 0.003 |
| SRL - Env Structuring | 6.06 | 2.00, 180.00 | .003 | .014 | 0.063 |
| SRL - Help Seeking | 7.89 | 2.00, 180.00 | <.001 | .003 | 0.081 |
| DEP Total | 4.73 | 2.00, 180.00 | .010 | .030 | 0.050 |
| DEP - Cognitive-Behavioral | 2.63 | 1.81, 162.88 | .081 | .161 | 0.028 |
| DEP - Functional Use | 1.21 | 2.00, 180.00 | .301 | .301 | 0.013 |
| DEP - Emotional | 4.15 | 2.00, 180.00 | .017 | .052 | 0.044 |
| Writing Performance (Total) | 2.32 | 2.00, 180.00 | .101 | .101 | 0.025 |
| Writing - Content | 0.89 | 1.68, 150.98 | .396 | .570 | 0.010 |
| Writing - Organization | 1.81 | 1.70, 153.34 | .172 | .570 | 0.020 |
| Writing - Vocabulary | 1.29 | 2.00, 180.00 | .278 | .570 | 0.014 |
| Writing - Language Use | 3.48 | 1.74, 156.90 | .039 | .197 | 0.037 |
| Writing - Mechanics | 2.01 | 1.76, 157.97 | .143 | .570 | 0.022 |

Greenhouse–Geisser correction is incorporated in df and p when Mauchly p < .05. Holm correction is a retrospective sensitivity analysis, separately across three totals, six SRL dimensions, three dependency dimensions, and five writing dimensions. These families were not preregistered.

# Table S7 Full time-specific group contrasts

| **Outcome** | **Time** | **SE** | ***t*** | ***df*** | **Unadjusted *p*** | **Bonferroni-adjusted *p*** |
| --- | --- | --- | --- | --- | --- | --- |
| SRL Total | T1 | 0.102 | -0.15 | 152.7 | .879 | 1.000 |
| SRL Total | T2 | 0.102 | 2.12 | 152.7 | .036 | .108 |
| SRL Total | T3 | 0.102 | 2.02 | 152.7 | .045 | .136 |
| SRL - Goal Setting | T1 | 0.131 | 0.12 | 220.6 | .905 | 1.000 |
| SRL - Goal Setting | T2 | 0.131 | 1.56 | 220.6 | .120 | .361 |
| SRL - Goal Setting | T3 | 0.131 | 1.31 | 220.6 | .193 | .579 |
| SRL - Task Strategy | T1 | 0.125 | 0.48 | 187.3 | .634 | 1.000 |
| SRL - Task Strategy | T2 | 0.125 | 2.15 | 187.3 | .033 | .098 |
| SRL - Task Strategy | T3 | 0.125 | 2.18 | 187.3 | .030 | .091 |
| SRL - Time Mgmt | T1 | 0.130 | 0.45 | 204.7 | .654 | 1.000 |
| SRL - Time Mgmt | T2 | 0.130 | 1.80 | 204.7 | .073 | .220 |
| SRL - Time Mgmt | T3 | 0.130 | 0.41 | 204.7 | .685 | 1.000 |
| SRL - Self Eval | T1 | 0.123 | 0.41 | 213.2 | .679 | 1.000 |
| SRL - Self Eval | T2 | 0.123 | 1.19 | 213.2 | .234 | .703 |
| SRL - Self Eval | T3 | 0.123 | 0.80 | 213.2 | .423 | 1.000 |
| SRL - Env Structuring | T1 | 0.134 | -1.04 | 209.8 | .297 | .892 |
| SRL - Env Structuring | T2 | 0.134 | 1.85 | 209.8 | .065 | .196 |
| SRL - Env Structuring | T3 | 0.134 | 2.64 | 209.8 | .009 | .027 |
| SRL - Help Seeking | T1 | 0.131 | -1.61 | 212.2 | .109 | .328 |
| SRL - Help Seeking | T2 | 0.131 | 1.50 | 212.2 | .136 | .408 |
| SRL - Help Seeking | T3 | 0.131 | 2.72 | 212.2 | .007 | .021 |
| DEP Total | T1 | 0.053 | -1.58 | 178.4 | .116 | .348 |
| DEP Total | T2 | 0.053 | -4.32 | 178.4 | <.001 | <.001 |
| DEP Total | T3 | 0.053 | -4.12 | 178.4 | <.001 | <.001 |
| DEP - Cognitive-Behavioral | T1 | 0.067 | -0.96 | 206.6 | .337 | 1.000 |
| DEP - Cognitive-Behavioral | T2 | 0.067 | -2.92 | 206.6 | .004 | .012 |
| DEP - Cognitive-Behavioral | T3 | 0.067 | -3.33 | 206.6 | .001 | .003 |
| DEP - Functional Use | T1 | 0.070 | -1.47 | 236.2 | .143 | .428 |
| DEP - Functional Use | T2 | 0.070 | -2.77 | 236.2 | .006 | .018 |
| DEP - Functional Use | T3 | 0.070 | -3.30 | 236.2 | .001 | .003 |
| DEP - Emotional | T1 | 0.067 | -1.27 | 227.6 | .204 | .613 |
| DEP - Emotional | T2 | 0.067 | -4.67 | 227.6 | <.001 | <.001 |
| DEP - Emotional | T3 | 0.067 | -3.02 | 227.6 | .003 | .009 |
| Writing Performance (Total) | T1 | 1.199 | 0.74 | 159.0 | .462 | 1.000 |
| Writing Performance (Total) | T2 | 1.199 | 2.65 | 159.0 | .009 | .027 |
| Writing Performance (Total) | T3 | 1.199 | 2.01 | 159.0 | .046 | .137 |
| Writing - Content | T1 | 0.370 | 0.49 | 179.4 | .627 | 1.000 |
| Writing - Content | T2 | 0.370 | 1.54 | 179.4 | .126 | .379 |
| Writing - Content | T3 | 0.370 | 1.72 | 179.4 | .087 | .260 |
| Writing - Organization | T1 | 0.268 | 0.78 | 197.9 | .439 | 1.000 |
| Writing - Organization | T2 | 0.268 | 2.81 | 197.9 | .005 | .016 |
| Writing - Organization | T3 | 0.268 | 1.78 | 197.9 | .077 | .231 |
| Writing - Vocabulary | T1 | 0.275 | 0.80 | 161.1 | .428 | 1.000 |
| Writing - Vocabulary | T2 | 0.275 | 2.26 | 161.1 | .025 | .075 |
| Writing - Vocabulary | T3 | 0.275 | 1.56 | 161.1 | .120 | .359 |
| Writing - Language Use | T1 | 0.314 | 0.63 | 174.2 | .531 | 1.000 |
| Writing - Language Use | T2 | 0.314 | 3.18 | 174.2 | .002 | .005 |
| Writing - Language Use | T3 | 0.314 | 2.17 | 174.2 | .031 | .093 |
| Writing - Mechanics | T1 | 0.076 | 1.05 | 183.7 | .294 | .882 |
| Writing - Mechanics | T2 | 0.076 | 3.04 | 183.7 | .003 | .008 |
| Writing - Mechanics | T3 | 0.076 | 2.44 | 183.7 | .016 | .047 |

Estimates, familywise 95% confidence intervals, and pooled-SD Cohen’s d are in main Tables 1 and 2. Model: score ~ Group * Time + (1 | participant), fitted by REML. Degrees of freedom (df) were estimated using the Kenward–Roger method. Bonferroni adjustment covers the three time-specific group contrasts within each outcome.

# Table S8 Dependency construct and data-column mapping

| **Published factor** | **Published items** | **Dataset columns** | **Interpretation** |
| --- | --- | --- | --- |
| Cognitive-Behavioral | Q1–Q6 | DEP_CB_1–6 | Priority use, central-tool reliance, cognitive and behavioral displacement |
| Functional | Q7–Q12 | DEP_FU_1–6 | Perceived utility, efficiency, task support and knowledge resources |
| Emotional | Q13–Q17 | DEP_EM_1–5 | Disclosure, emotional understanding, satisfaction and mood regulation |

Source: Yu and Yan (2026), Table 8, p. 89. The published instrument contains 17 positively scored five-point items. The total sum ranges from 17 to 85; the reported total mean equals the sum divided by 17. The correspondence above identifies factor membership and sequential column labels. The questionnaire was administered without changes to the published item wording. No new factor-validation claim is made.

# Table S9 Current-sample internal consistency

| **Scale or dimension** | **Items** | **α T1** | **α T2** | **α T3** |
| --- | --- | --- | --- | --- |
| SRL GS | 3 | 0.624 | 0.578 | 0.690 |
| SRL TS | 4 | 0.689 | 0.592 | 0.719 |
| SRL TM | 4 | 0.697 | 0.639 | 0.719 |
| SRL SE | 4 | 0.670 | 0.634 | 0.583 |
| SRL ES | 3 | 0.615 | 0.541 | 0.712 |
| SRL HS | 3 | 0.602 | 0.588 | 0.683 |
| SRL Total | 21 | 0.903 | 0.891 | 0.910 |
| DEP CB | 6 | 0.608 | 0.637 | 0.733 |
| DEP FU | 6 | 0.702 | 0.671 | 0.738 |
| DEP EM | 5 | 0.623 | 0.647 | 0.617 |
| DEP Total | 17 | 0.783 | 0.806 | 0.843 |

Cronbach’s α was calculated separately at each assessment using all 92 completers. No reverse scoring was applied. α does not verify dimensionality or measurement invariance.

# Table S10 Complete between-group LSA comparisons

| **Transition** | **Agent CTP** | **AMP CTP** | **z** | **Unadjusted *p*** | **Bonferroni-adjusted *p*** |
| --- | --- | --- | --- | --- | --- |
| PLAN → PLAN | 0.034 | 0.031 | 0.14 | .886 | 1.000 |
| PLAN → MON | 0.359 | 0.356 | 0.07 | .947 | 1.000 |
| PLAN → REV | 0.184 | 0.105 | 2.25 | .025 | .492 |
| PLAN → PASS | 0.398 | 0.497 | -1.99 | .047 | .933 |
| PLAN → REFL | 0.024 | 0.010 | 1.04 | .297 | 1.000 |
| MON → PLAN | 0.019 | 0.018 | 0.03 | .977 | 1.000 |
| MON → MON | 0.308 | 0.262 | 0.98 | .326 | 1.000 |
| MON → REV | 0.117 | 0.079 | 1.20 | .229 | 1.000 |
| MON → PASS | 0.234 | 0.329 | -2.06 | .039 | .782 |
| MON → REFL | 0.322 | 0.311 | 0.24 | .813 | 1.000 |
| REV → PLAN | 0.045 | 0.019 | 0.82 | .415 | 1.000 |
| REV → MON | 0.202 | 0.283 | -1.10 | .270 | 1.000 |
| REV → REV | 0.112 | 0.132 | -0.35 | .726 | 1.000 |
| REV → PASS | 0.348 | 0.189 | 2.03 | .042 | .847 |
| REV → REFL | 0.292 | 0.377 | -1.05 | .294 | 1.000 |
| PASS → PLAN | 0.013 | 0.004 | 1.16 | .245 | 1.000 |
| PASS → MON | 0.246 | 0.143 | 2.90 | .004 | .074 |
| PASS → REV | 0.070 | 0.049 | 1.00 | .315 | 1.000 |
| PASS → PASS | 0.285 | 0.398 | -2.64 | .008 | .165 |
| PASS → REFL | 0.386 | 0.406 | -0.45 | .650 | 1.000 |

Adjusted p values use Bonferroni correction across 20 cells. These aggregate comparisons do not account for participant-level dependence and remain exploratory.

| **Origin** | **χ²** | ***df*** | **Unadjusted *p*** | **Bonferroni-adjusted p¹** | **Min E** | **E < 5** |
| --- | --- | --- | --- | --- | --- | --- |
| PLAN | 7.60 | 4 | .107 | .429 | 3.37 | 2 |
| MON | 5.12 | 4 | .276 | 1.000 | 3.04 | 2 |
| REV | 5.36 | 4 | .252 | 1.000 | 1.87 | 2 |
| PASS | 13.79 | 4 | .008 | .032 | 1.85 | 2 |

¹ Bonferroni correction across four originating rows. E = expected cell count. Summed nominal omnibus χ²(16) = 31.86, p = .010; this aggregate test is exploratory. One REFL → PASS event in AMP is outside the four-origin table.
